# White matter hyperintensities in bipolar disorder: systematic review and meta-analysis

**DOI:** 10.3389/fpsyt.2024.1343463

**Published:** 2024-01-26

**Authors:** Tânia Silva, Cesar Nunes, Andreia Ribeiro, Isabel Santana, Joaquim Cerejeira

**Affiliations:** ^1^ Department of Psychiatry, Centro Hospitalar e Universitário de Coimbra, Coimbra, Portugal; ^2^ Faculty of Medicine, University of Coimbra, Coimbra, Portugal; ^3^ Coimbra Institute for Clinical and Biomedical Research (iCBR), Faculdade de Medicina da Universidade de Coimbra, Coimbra, Portugal; ^4^ Department Neuroradiology, Centro Hospitalar e Universitário de Coimbra, Coimbra, Portugal; ^5^ Department of Neurology, Centro Hospitalar e Universitário de Coimbra, Coimbra, Portugal

**Keywords:** bipolar disorder, mood disorder, cerebral small vessel disease, cerebrovascular disease, microvascular disease, white matter hyperintensities, white matter lesions

## Abstract

**Background:**

White matter hyperintensities are lesions of presumed vascular origin associated with Cerebral small vessel disease. WMH are common findings that and are associated with increased risk of cognitive impairment and dementia. A higher prevalence of WMH has been also reported in patients with bipolar disorder (BD), although the evidence is conflicting.

**Objective:**

To compare the prevalence of WMH in adults with BD, with the prevalence found in healthy controls.

**Methods:**

We searched the Embase, Medline/PubMed, and references cited in articles retrieved on May 20, 2023. We included case-control studies that compared the prevalence of WMH in adult BD patients with the prevalence of WMH in healthy controls, using T2-weighted magnetic resonance imaging. We performed a meta-analysis using a random-effects method based on the inverse-variance approach.

**Findings:**

We included 22 case-control studies reporting data of 1313 people. The overall rate of WMH was 46.5% in BD patients and 28% in controls (pooled Odds Ratio 2.89, 95% CI 1.76; 4.75). We found a moderate heterogeneity across studies (I^2 =^ 0.49). Publication bias was not significant.

**Interpretation:**

We found evidence that BD patients have a higher burden of WMH than healthy controls. Main limitations were impossibility of analyzing gender differences and bipolar type, moderate heterogeneity between studies, non-representative samples, lack of control for major confounders and search in two electronic databases.

**Systematic review registration:**

https://www.crd.york.ac.uk/prospero/display_record.php?ID=CRD42023428464

## 1 Introduction

White matter hyperintensities (WMH) are a common finding in every-day practice of psychiatrists and neurologists. WMH are defined as hyperintense signs in the brain tissue that appear in the T2-weighted (T2W) and fluid-attenuated inversion recovery (FLAIR) sequences of magnetic resonance imaging (MRI). These neuroimaging findings correspond to white matter lesions of presumed vascular origin due to cerebral small vessel disease (CSVD). Because Cerebral small vessels cannot be visualized *in vivo*, WMH are used as a biomarker of CSVD (1, 2).

CSVD refers to a group of pathological processes with multiple etiologies leading to damage of small cerebral vessels. Mechanisms involved include chronic ischemia, hemorrhage, blood-brain barrier damage, oligodendrocytes apoptosis and inflammation (2). It has been suggested that these processes induce pathological changes such as narrowing and abnormal motor regulation of small cerebral vessels due to lipohyalinosis and fibrohyalinosis (3). The most common CSVD is sporadic and has a strong association with age and cerebrovascular risk factors, especially hypertension (2–4).

The prevalence of WMH increases with age, ranging from 5% in people aged 50 years, to 100% in people aged 90 or older (3, 4). Some studies found a higher prevalence and severity in women (1, 4). The presence of WHM has been consistently linked to cognitive and psychiatric disturbances, particularly the development of cognitive impairment and dementia (1).

Studies investigating WMH in Bipolar patients suggested a possible association between Bipolar Disorder (BD) and CSVD, although the results are conflicting (2). As with CSVD, BD patients have higher burden of vascular risk factors (5). The association between BD and vascular risk factors has been traditionally attributed to psychopharmacological treatments and/or medical comorbidities. However, the strength of this association is higher than the expected (5). Thus, BD patients manifest cardiovascular disease earlier than the general population and have higher mortality rates from vascular events than the general population. This increased rate of cardiovascular morbidity and mortality is reported in the literature prior to the use of mood stabilizers and antipsychotics (5). These findings raised the hypothesis of a bi-directional relationship between BD and vascular disease. Alternatively, BD and vascular disease may have a shared underlying cause (5–7).

The terms “poststroke mania” and “vascular mania” have been used in bipolar patients when a manic episode is thought to be the consequence of a prior vascular event (7). Features that favor this diagnosis include: late onset of symptoms or modification of the course of the disease after the age of 50, absence of a family history and marked functional disability (7, 8).

The validation of “vascular mania” as a clinical-pathological entity would have a significant impact in clinical practice. This subtype of mania would have a different clinical presentation, evolution, and response to treatment, when compared to non-vascular BD.

Clarifying the relationship between BD and CSVD can potentially provide new insights regarding the pathophysiology of BD, and allow the development of new interventions capable of modifying the course of BD.

Therefore, estimating the prevalence of WMH in BD patients is an important objective from both scientific and clinical perspective. The last meta-analysis was carried out in 2009 by Beyer JL et al. and reported an odds ratio of 2.5 (95% CI 1.9, 3.3) for hyperintensities in BD patients compared to controls (9). Meanwhile, other studies using more recent MRI technology have been published.

The aim of this study is to estimate the prevalence of WMH in patients with BD. For this purpose, we performed a systematic review with meta-analysis.

## 2 Methodology

### 2.1 Search strategy

**Figure 1:**
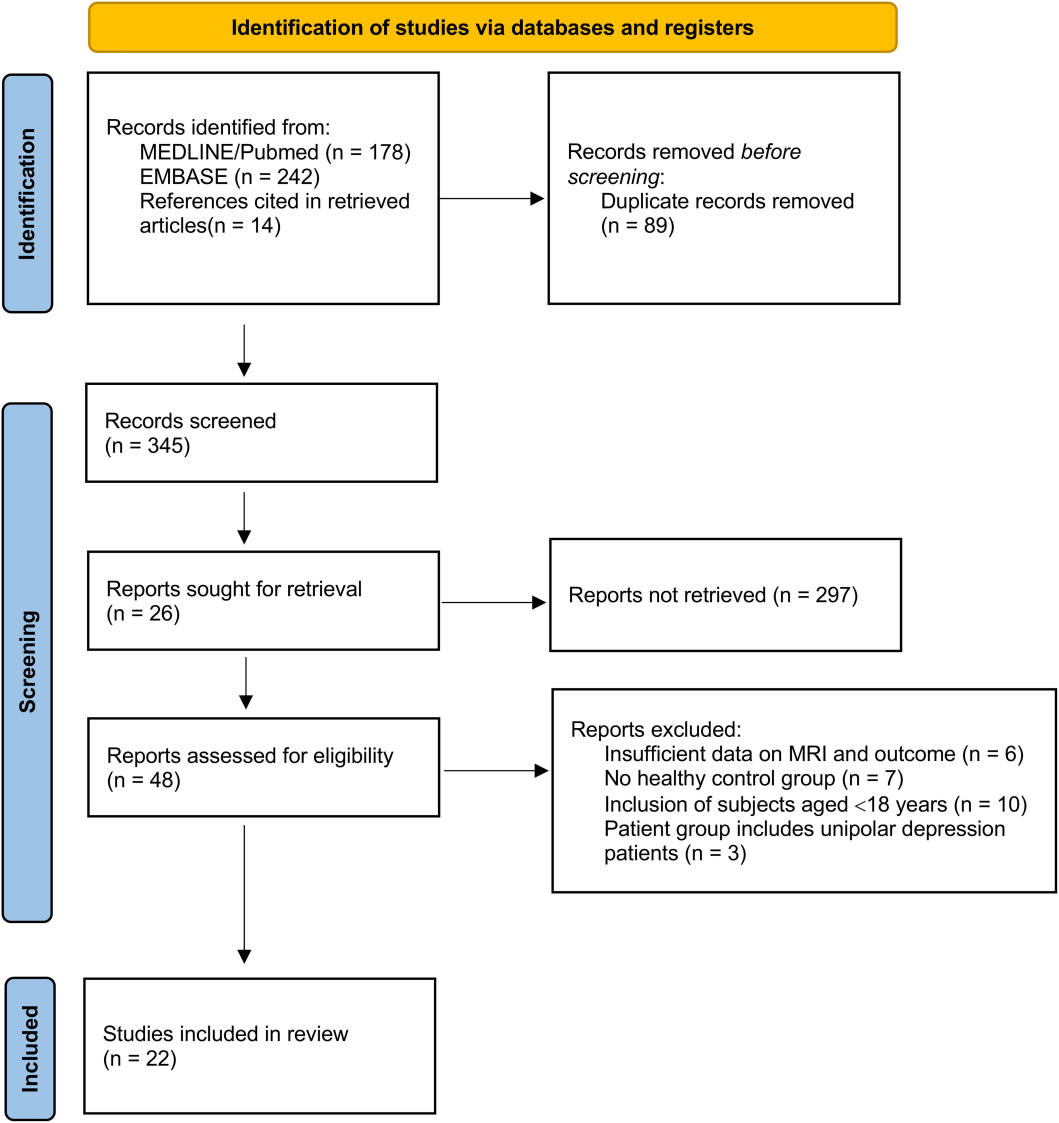
PRISMA flow diagram for study selection (10).

We followed the PRISMA statement, flow chart and checklist to develop this systematic review and meta-analysis (**Figure 1**).

We conducted an electronic search on Embase, Medline/PubMed and reviewed the references cited in retrieved articles on 20 May 2023. We didn’t use any restrictions regarding country, race or sex. Language was restricted to literature written in English, Portuguese, and Spanish.

The search strategy included the combination of terms related to the PEO framework (**Supplementary material**). The keywords used for the search were: “White matter hyperintensities”, “Cerebral Small Vessel Diseases”, “microvascular disease”, “leukoaraiosis”, “Leukoencephalopathy”, “bipolar disorder”, “mania” and “hypomania”.

PROSPERO was searched to ensure a similar systematic review study protocol has not been registered. No prior studies on our topic of interest have been identified. PROSPERO registration number: 428464.

### 2.2 Study selection

The studies were selected using the following inclusion criteria: a) participants aged ≥18 years old; b) BD diagnosis based on the criteria of the Diagnostic and Statistical Manual of Mental Disorders (DSM) or the International Statistical Classification of Diseases and Related Health Problems (ICD) classifications; c) case-control studies using healthy participants as the control group; d) assessment of WMH with T2-weighted magnetic resonance imaging; e) studies evaluating all the participants according to identical MRI outcomes and providing the proportion of BD patients and healthy controls affected by WMH. PROSPERO protocol requirement “Same MRI acquisition conditions used for all subjects of the study” was applied by including studies that assessed WMH with T2-weighted acquisitions.

We excluded studies in which the control group included subjects with a family relationship with the patients with BD; when WMH assessment method was inadequate, or data were unreliably extracted; duplicate or overlapping data and articles without available full text.

### 2.3 Procedure and data extraction

All the records retrieved by the electronic search were downloaded into a bibliographic manager (Mendeley^®^ Desktop, version 1.19.8). After removing duplicates, two independent reviewers screened the titles, abstracts and full texts against the established selection criteria.

Data was extracted using a piloted form: first author and publication date, sample size and demographic characteristics, cardiovascular risk factors assessment, diagnostic criteria of BD (DSM or ICD), MRI details (type of scan, field strength and ponderation characteristics), measurement methods and WMH prevalence, and information for assessment of the risk of bias. TS and AR independently extracted the information. Differences were resolved by discussion, with the involvement of a third review author (JC). The excluded studies and the reason are presented in **Supplementary material**.

### 2.4 Quality and major confounders assessment

TS and AR independently assessed the risk of bias according to the latest quality assessment tool guide recommendations (11). TS and AR used a form checklist based on Newcastle-Ottawa quality assessment scale for case-control studies (**Table 1** and **Supplementary material**) (11). Differences were resolved by discussion, with the involvement of a third review author (JC). The following characteristics were considered: adequate case definition, representativeness of the cases, selection of controls, definition of controls, comparability of cases and controls based on the design or analysis, ascertainment of exposure, same method of ascertainment for cases and controls and non-response rate.

**Table 1:** Effect size estimates of all studies included in the meta-analysis.

	Exp. Effect size	Standard error	T	p-Value	95% CI Lower	95% CI upper
Total studies	2.883	0.248	4.269	<0.001	1.669	4.891

The methods used to control for major confounders were assessed. The following potential confounding factors were considered: age and sex, cardiovascular risk factors, psychiatric medication, substance abuse and medical/neurologic comorbidity (**Supplementary material**).

### 2.5 Effect measures and statistical analysis

Data were combined in a meta-analysis performed with IBM^®^SPSS^®^Statistics (version: 28.0.1.0) software.

**Figure 2:**
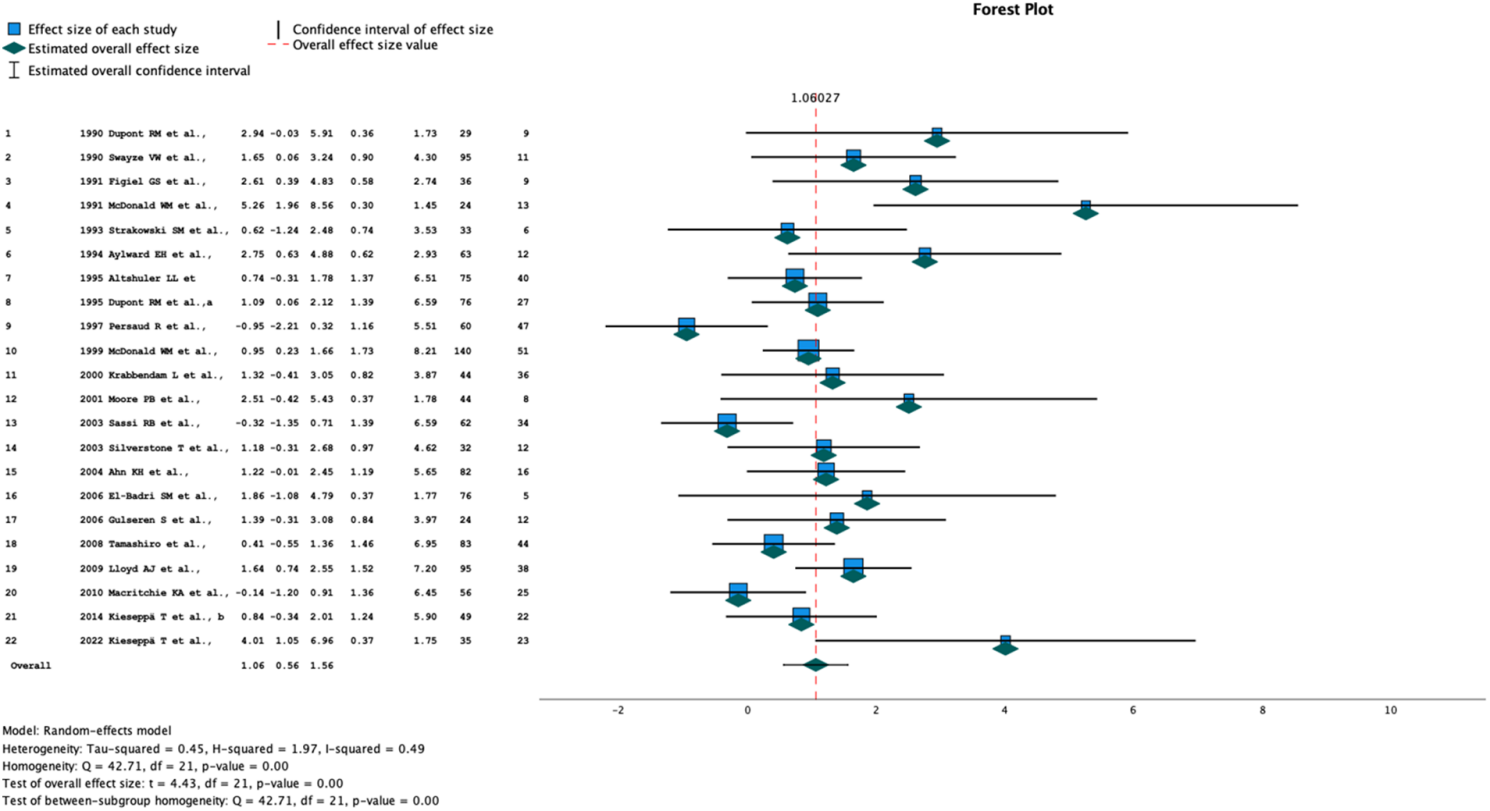
Forrest plot of all studies included in the meta-analysis. Columns - study number, year and author, effect size, lower and upper 95% CI, Weight (%), total number of participants, number of subjects with white matter hyperintensities.

We anticipated between-study heterogeneity, so a random-effects model was used to pool effect sizes. We used the number of subjects with WMH in the BD and control groups to calculate pooled odds ratios (OR). In addition to the overall meta-analysis, we also performed a separate meta-analysis of two groups of studies, those using 0.5T and those using 1.5T field strength. Heterogeneity between studies was estimated with the I^2^ statistic. We used Knapp-Hartung adjustments to calculate the confidence interval (CI) around the pooled effect. We performed a meta-regression analysis with the restricted maximum likelihood (REML) method to explore sources of heterogeneity. The meta-analysis is presented in a forest-plot (**Figure 2** and **Tables 1**, **2**).

**Table 2:** Characteristics of identified studies and prevalence of WMH in BD patients and healthy controls.

Study	Sample size	Mean age	Male/Female	Diagnostic criteria	MRI characteristics	WMH proportion
Dupont RM et al. (1990) (12)	BP=19 HC=10	BP: 36.5 HC: 41 (Age <55)	BP: 19/1 HC: all male	DSM-III	1.5T, GE Signa, T2W ST/IG: 5/2.5 TR/TE: 2000/ 25; 70	**WMH presence** BP:47%; HC:0%
Swayze VW et al. (1990) (13)	BP=48 HC=47	BP: 33.9 HC: 34.7	BP: 29/19 HC: 28/19	DSM-III and DSM-III-R	0.5T, Phillips Picker, T2W ST/IG: 10/0 TR/TE: 2000/20; 70	**WMH presence** BP: 18.8%; HC: 24.2%
Figiel GS et al. (1991) (14)	BP=18 HC=18	BP: 37.5 HC: 34.7 (age <60)	BP: 5/13 HC: 8/10	DSM-III	1.5T, GE Signa, T2W ST/IG: 5/2,5 TR/TE: 2500/40; 80	**DWMH presence** BP: 44.4%; HC: 5.6%
McDonald WM et al. (1991) (15)	BP=12 HC=12	BP: 68.3 ± 7 HC: 68.7 ± 7	BP: 6/6 HC: 6/6	DSM-III-R	1.5T, GE Signa, T2W ST/IG: 5/2,5 TR/TE: 2800/30; 80	**WMH presence** BP: 100%; HC: 75%
Strakowski SM et al. (1993) (16)	BP=18 HC=15	BP: 31.3 ± 11.8 HC: 32.4 ± 8.8 (age range 18-65)	BP: 8/10 HC: 7/8	DSM-III-R	1.5T, GE Signa, T2W ST/IG: 5/2,5 TR/TE: 2000/40; 80	**WMH presence** BP: 22.2%; HC: 13.3%
Aylward EH et al. (1994) (17)	BP=32 HC=31	BP: 39.3 ± 11.1 HC: 37.6 ± 9 (age range 20-59)	?	DSM-III-R	1.5T, GE Signa, T2W ST/IG: 5/_ TR/TE: 2500/80	**WMH presence** BP: 34.4%; HC: 3.2%
Altshuler LL et al. (1995) (18)	BP=55 (BPI and BPII) HC=20	BP: 40.8 HC: 35.2	BP: 29/26 HC: 11/9	SADS	0.5T, Philips Picker, T2W ST/IG: 10/_ TR/TE: 2000/30	**WMH presence** BP: 58.2%; HC: 40%
Dupont RM et al. (1995)^a^ (19)	BP=44 HC=32	BP:36.6 ± 10.7 HC:39.2 ± 8.9	BP: 28/16 HC: 19/13	DSM-III-R	1.5T, GE Signa, T2W? ST/IG: 5/2,5 TR/TE: 2000/25	**WMH presence** BP: 45.5%; HC: 21.9%
Persaud R et al. (1997) (20)	BP=26 HC=34	BP: 35.6 HC: 31.6 (age range: 19-49)	BP: 10/16 HC: 19/15	DSM-III-R	0.5T, Philips Picker, T2W ST/IG: _/_ TR/TE: 2400/70	**WMH presence** BP: 69.2%; HC: 85.3%
McDonald WM et al. (1999) (21)	BP=70 HC=70	BP: 49.4 HC: 53.2 (Age <60)	BP:30/40 HC:32/38	DSM-III-R	1.5T, GE Signa, T2W ST/IG: 5/2.5 TR/TE: 2800/30; 80	**PWMH presence** BP:31.4%; HC: 28.6% **DWMH presence** BP: 47.1%; HC: 25.7%
Krabbendam L et al. (2000) (22)	BP=22 HC=22 (age <60)	BP: 47.7 ± 8.3 HC: 41.4 ± 11.3	BP: 5/17 HC: 10/12	DSM-IV	1.5T, Philips Gyroscan, T2W ST/IG: 5/0.5 TR/TE: 3000/23; 120	**PWMH presence** BP:27.3%; HC:4.5% **DWMH presence** BP: 90%; HC: 72.7%
Moore PB et al. (2001) (23)	BP=29 HC=15	BP: 44.66 HC: 41.9 (age range 20-65)	BP: 15/14 HC: 7/8	DSM-IV	0.5T, GE MR max plus, T2W ST/IG: 7/1 TR/TE: 2300/25; 100	**DWMH presence** BP: 27.6%; HC:0% **PWMH presence** BP: 62%; HC: 46.7%
Sassi RB et al. (2003) (24)	BP=24 HC=38	BP: 34.2 ± 9.9 HC: 36.8 ± 9.7 (age range 19 to 59)	BP: 15/9 HC: 24/14	DSM-IV	1.5T, GE Signa, T2W ST/IG: 5/0 TR/TE: no data.	**WMH presence** BP: 50%; HC: 57.9%
Silverstone T et al. (2003) (25)	BP=13 HC=19	BP: 40.2 HC: 35.9 (age range 19-65)	BP: 7/6 HC: 9/10	DSM-III-R and DSM-IV	0.5T, Philips?, T2W ST/IG: 5/2.5 TR/TE: no data.	**WMH presence (Fazekas score** **2 or 3)** BP:53.8%; HC:26.3%
Ahn KH et al. (2004) (26)	BP=43 HC=39	BP: 36.9 ± 11.5 HC: 35.1 ± 9.7 (age range 18 to 59)	BP: 19/24 HC: 17/22	DSM-IV	1.5T, GE Signa, T2W ST/IG: 5/2 TR/TE: 3000/30; 80	**WMH presence** BP: 27.9%; HC: 10.3%
El-Badri SM et al. (2006) (27)	BP=50 HC=26	BP: 30.2 ± 6.2 HC: 30.2 ± 6.2	BP: 15/35 HC: 13/13	DSM-IV	0.5T, GE MR max plus, T2W ST/IG: 7/1 TR/TE: 2300 TE:25/100	**DWMH presence** BP: 10%; HC: 0%
Gulseren S et al. (2006) (28)	BP=12 HC=12	BP: 30.9 ± 3.6 HC: 30.4 ± 3.6 (aged ≤45)	BP: 8/4 HC: 8/4	DSM-IV	0.5T, GE Vectra, T2W ST/IG: 5/7 TR/TE: 2000/90	**WMH presence** BP: 66.7%; HC: 33.3% **PWMH presence** BP: 0%; HC: 0% **DWMH presence** BP: 16.6%; HC: 8.3%
Tamashiro et al. (2008) (29)	BP=59 HC=24	BP: 68.76 ± 4.87 HC: 69 ± 7.22 (aged ≥60)	BP: 20/39 HC: 6/18	DSM-IV and ICD-10	1.5T, GE Signa, T2W ST/IG: 6/0.6 TR/TE: 4000/98	**WMH presence** Frontal PWMH - BP: 54.2%; HC: 33.3% Frontal DWMH - BP: 55.9%; HC: 45.8% Parietal DWMH - BP: 44%; HC: 29.1%
Lloyd AJ et al. (2009) (30)	BP=48 HC=47	BP: 44.5 ± 8.9 HC: 45.8 ± 8.3	BP: 22/26 HC: 19/28	DSM-IV	1.5T, GE Signa, T2W ST/IG: 5/2 TR/TE: 3420/97.2 FLAIR ST/IG: 5/2 TR/TE/IT: 10002/142/2100	**WMH presence** PVWMH – BP: 20.8%; HC: 8.5% DWMH – BP: 58.3%; HC: 42.6%
Macritchie KA et al. (2010) (31)	BP=28 HC=28	BP: 43 ± 11.5 HC: 43 ± 11.7	BP: 16/12 HC: 16/12	DSM-IV-TR	1.5T, GE Signa, T2W ST/IG: 5/_ TR/TE: 5000/102	**DWMH presence** BP: 42.9%; HC: 46.4% **PWMH presence** BP: 39.3%; HC: 25%
Kieseppä T et al. (2014) (32)	BP=28 HC=21	BP: 40.4 HC: 41.7	BP: 17/11 HC: 10/11	DSM-IV	1.5T, Siemens Magnetom, T2W ST/IG: 5/1 TR/TE: 5300/112 FLAIR ST/IG: 5/1 TR/TE/IT: 10000/148/?	**DWMH presence** BP: 53.6%; HC: 33.3% **PWMH presence** BP: 42.8%; HC: 61.9%
Kieseppä T et al. (2022) (33)	BP=16 HC=19	BP: 45.85 HC: 49.6	BP: 9/7 HC: 8/11	DSM-IV	*Follow-up assessment:* 3T, Philips Achieva, T2W ST/IG: 5/0.5 TR/TE: 4000/80 FLAIR ST/IG: 5/0.5 TR/TE/IT: 11000/120/?	**WMH presence at follow-up assessment:** BP:100%; HC:36.8%

M, male; F, female; VRF, Cardiovascular risk factors; White Matter Hyperintensities, ST/IG, slice thickness/interscan gap; TR/TE, repetition time/echo time; TR/TE/IT, repetition time/echo time/inversion time; BP, bipolar patients; HC, healthy control; WM, white matter; WMH, white matter hyperintensities; PWMH, periventricular white matter hyperintensities; DWMH, deep white matter hyperintensities; SADS, Schedule for Affective Disorder and Schizophrenia; a October 1995.

## 3 Results

### 3.1 Results of research

A flow diagram with the selection of the studies is shown in **Figure 1**. The search strategy produced 434 titles, of which 213 were discarded by title and abstract (71 case reports, 13 written in non-eligible language, 213 inadequate study design or intervention) and 89 for duplicity. Of these, 26 were excluded according to the exclusion criteria. In total, we included 22 studies for meta-analysis.

### 3.2 Included studies

The 22 selected case-control studies are presented in **Table 2** (12–29, 31–33). The included studies were published from 1990 to 2022.

### 3.3 Participants

The total number of participants is 1313, with mean ages ranging from 30 to 69 years. Sample sizes are generally small, ranging from 24 to 140 participants. Overall, the studies included 714 patients with BD and 599 healthy controls. In all studies, age match was performed between groups. Sex match was done in all but two studies. In the studies from Dupont RM et al. (12) and Aylward EH et al. (17) the sex of participants was not reported so an overall ratio could not be obtained.

Matching for cardiovascular risk factors was done in 7 studies (14, 21, 22, 25, 29, 30, 32). In Krabbendam L et al. (32), Silverstone T et al. (25), Lloyd AJ et al. (30) and Kieseppä T et al. (32) participants with cardiovascular risk factors were excluded.

The criteria for BD used in the studies were SADS, DSM-III, DSM-III-R, DSM-IV, DSM-IV-TR and ICD-10. While some studies specify the inclusion of BD type I and II patients, most do not refer to the subtype. Some studies included schizophrenia and depressive patients, but we were able to extract data from BD patients. Most studies did not specify the phase of the disease.

### 3.4 MRI characteristics

Seven studies used a field strength of 0.5-Tesla, 14 studies used a field strength of 1.5-T and in one study a field strength of 3-T was used.

The ponderation, slide thickness and interslice gap characteristics used in MRI acquisition were heterogeneous across studies and some of the studies did not provide complete data.

In 12 studies the General Electrics Signa scanner was used, 3 studies used the Philips Picker scanner, 2 studies used the General Electrics MR Max scanner and the rest used the Philips Gyroscan, the General Electrics Vectra, the Philips Achieva and the Siemens Magnetom scanner. In one study (25) the scanner specific model was not identified.

### 3.5 Prevalence of hyperintensities

WMH is identified as high signal regions on T2-weighted images. The studies reported proportions, or the total count of subjects affected by WMH.

The overall rate of WMH was 46.5% in BD patients and 28% in controls. Eight studies reported prevalence data with a significant difference between groups (13–15, 17, 19, 21, 30, 33). Fourteen studies reported no significant difference, but a trend toward increased prevalence of WMH in BD patients (12, 16, 18, 20, 22–29, 31, 33).

Half of the studies accessed the severity by measuring the size and confluence, or with visual rating scales, including Scheltens scale, Fazekas scale, Coffey scale and Boyko scale. The differences between the rating procedures precludes a combined analysis.

The summary OR estimate was 2.89 (95% CI 1.76, 4.75) (**Figure 2** and **Tables 1**, **2**). Subgroup analysis of studies using a 0.5-T field strength showed no significant difference between BD patients and controls. Subgroup analysis of the studies using a 1.5-T field strength showed a significant difference between BD patients and controls: the pooled OR estimate was 2.8 (95% CI 1.53, 5.14).

### 3.6 Location of hyperintensities

Few studies provided data on lesion location. The location was reported according to periventricular and deep white matter, lobar topography, and hemisphere. In studies reporting topography of the lesions, the frontal lobe was the most affected (14, 17, 22, 28–30), followed by fronto-parietal location (14, 17, 28). Only 2 studies reported laterality, in one study the left hemisphere was more affected (30) and in the other (28) the right hemisphere was the most affected.

### 3.7 Excluded studies

Twenty-six studies were excluded from the review due to insufficient data on the outcome or MRI (n = 6), due to lack of healthy control group (n = 7), due to the inclusion of subjects under the age of 18 years (n = 10) and due to the inclusion of patients with unipolar depression (n = 3).

### 3.8 Sources of heterogeneity

The heterogeneity test suggested moderate heterogeneity between studies (I^2 =^ 0.49). A meta-regression analysis revealed that sample size (p=0.432), publication year (p=0.541), mean age (p=0.559) and field strength (0.5T p=0.104, 1.5T p= 0.117) were not significant sources of heterogeneity (**Supplementary material**).

### 3.9 Risk of bias

**Figure 3:**
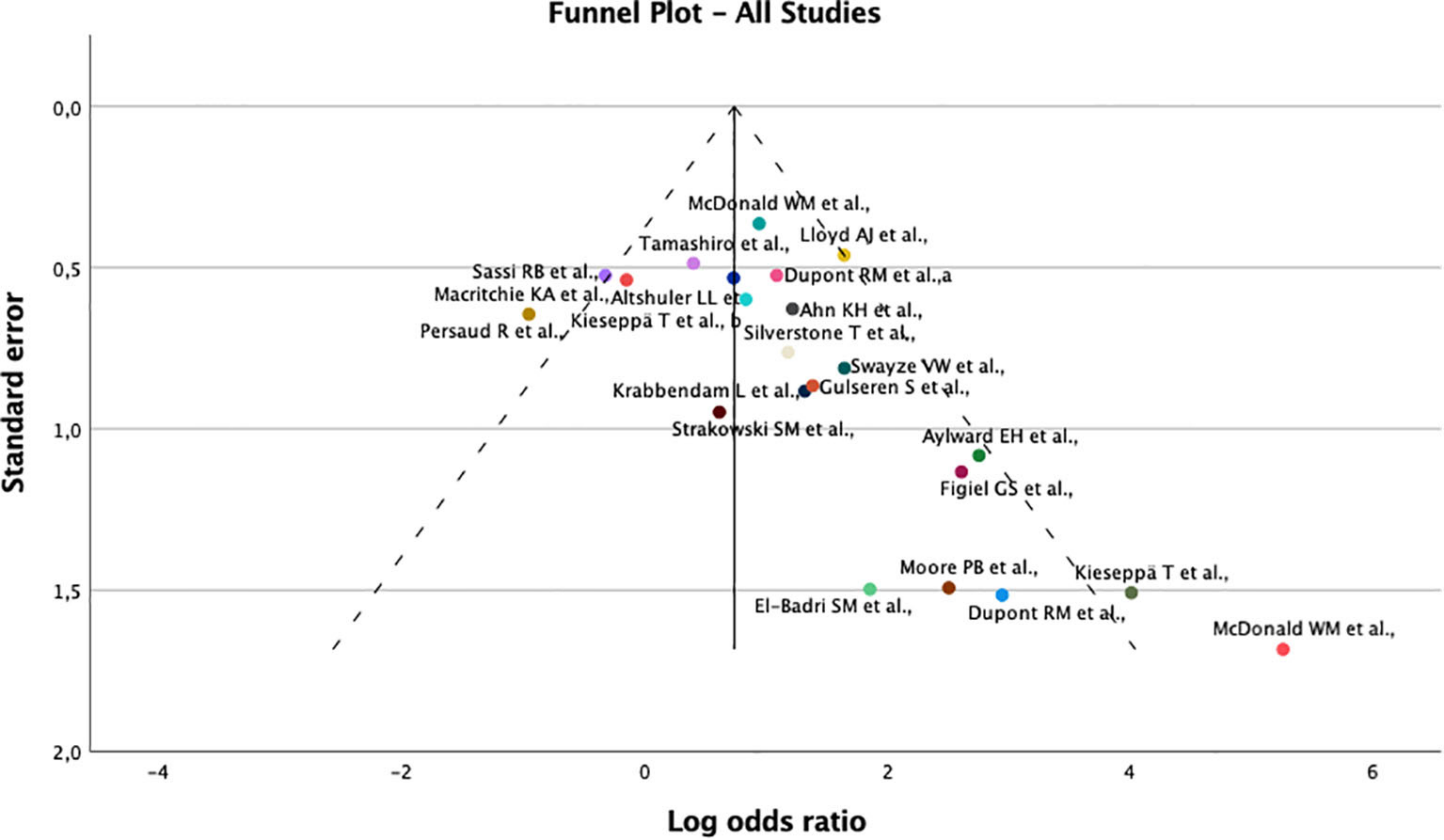
Funnel plot used to assess publication bias among the included studies.

The risk of bias assessment was performed using the Newcastle Ottawa Scale for case-control studies (**Table 3** and **Supplementary material**). The score ranged from 3 to 5, corresponding to fair quality studies. A funnel plot was used to assess publication bias among the included studies (**Figure 3**). Despite the apparent asymmetry, the Egger’s test found no evidence of significant publication bias (t=-1.976; 95% CI 3.776, 0.108, p=0.063).

**Table 3:** Critical appraisal using NewCastle-Ottawa Scale for case-control studies.

Study	Selection				Comparability	Exposure			Total
Dupont RM et al. (1990) (12)				*		*	*		**3**
Swayze VW et al. (1990) (13)				*	*	*	*		**4**
Figiel GS et al. (1991) (14)				*	*	*	*		**4**
McDonald WM et al. (1991) (15)				*	*	*	*		**4**
Strakowski SM et al. (1993) (16)				*	*	*	*		**4**
Aylward EH et al. (1994) (17)				*		*	*		**3**
Altshuler LL et al. (1995) (18)		*		*	*	*	*		**5**
Dupont RM et al. (1995) (19)				*	*	*	*		**4**
Persaud R et al. (1997) (20)				*	*	*	*		**4**
McDonald WM et al. (1999) (21)				*	*	*			**3**
Krabbendam L et al. (2000) (22)				*	*	*	*		**4**
Moore PB et al. (2001) (23)				*	*	*	*		**4**
Sassi RB et al. (2003) (24)				*	*	*	*		**4**
Silverstone T et al. (2003) (25)				*	*	*	*		**5**
Ahn KH et al. (2004) (26)				*	*	*	*		**5**
El-Badri SM et al. (2006) (27)				*	*	*	*		**4**
Gulseren S et al. (2006) (28)				*	*		*		**3**
Tamashiro et al. (2008) (29)				*	*	*	*		**4**
Lloyd AJ et al. (2009) (30)				*	*	*	*		**4**
Macritchie KA et al. (2010) (31)				*	*	*	*		**4**
Kieseppä T et al. (2014) (32)			*	*	*	*	*		**5**
Kieseppä T et al. (2022) (33)			*	*	*	*	*		**5**

### 3.10 Major confounders

All studies dealt with the confounding effect of age by matching groups for this factor. In all studies, except for Dupont RM et al. (1990) (12) and Aylward EH et al. (1994) (17) the groups were matched for sex.

To reduce the potential confounding effect of CVRF, Figiel GS et al. (1991) (14) and Tamashiro et al. (2008) (29) demonstrated a balance between groups for the confounder, showing a similar incidence of CVRF between groups. In Dupont RM et al. (1995) (19), Persaud R et al. (1997) (20), Krabbendam L et al. (2000) (22), Silverstone T et al. (2003) (25), Gulseren S et al. (2006) (28), Lloyd AJ et al. (2009) (30), Kieseppä T et al. (2014) (32) and Kieseppä T et al. (2022) (33) the authors restricted the selection of subjects with CVRF. Dupont RM et al. (1995) (19), Persaud R et al. (1997) (20) and Gulseren S et al. (2006) (28) excluded hypertensive subjects; Krabbendam L et al. (2000) (22) excluded diabetic and hypertensive patients; in Silverstone T et al. (2003) (25) none of the subjects had diabetes or cardiovascular disease; Lloyd AJ et al. (2009) (30), Kieseppä T et al. (2014) (32) and Kieseppä T et al. (2022) (33) excluded subjects with cardiovascular disease and hypertension.

The confounding effect of psychiatric medication was only accounted in Strakowski SM et al. (1993) (16) and Sassi RB et al. (2003) (24). Strakowski SM et al. (1993) (16) excluded subjects that had taken antipsychotic medication and Sassi RB et al. (2003) (24) excluded subjects taking any psychotropic drug rather that lithium.

For controlling the confounding effect of substance abuse Dupont RM et al. (1990) (12), Aylward EH et al. (1994) (17), Dupont RM et al. (1995)(29), Persaud R et al. (1997) (20), Krabbendam L et al. (2000) (22), Moore PB et al. (2001) (23), Sassi RB et al. (2003) (24), Ahn KH et al. (2004) (26), El-Badri SM et al. (2006) (27), Gulseren S et al. (2006) (28), Lloyd AJ et al. (2009) (30), Macritchie KA et al. (2010) (31), Kieseppä T et al. (2014) (32) and Kieseppä T et al. (2022) (33) excluded the selection of substance users. Tamashiro et al. (2008) (29) controlled for this factor by demonstrating a similar prevalence of substance abuse between groups.

All studies, except Swayze VW et al. (1990) (13), Persaud R et al. (1997) (20) and McDonald WM et al. (1999) (21), controlled for the potential confounding effect of medical/neurological diseases by restricting the selection of subjects with history these comorbidities.

## 4 Discussion

In this study we performed a systematic review and meta-analysis to estimate the prevalence of WMH in patients with BD. We included 22 studies which recruited 1313 participants, 714 with BD. The aggregated prevalence of WMH in participants with BD was 46.5% which is 2.89-fold higher than in controls.

### 4.1 Cerebral small vessels disease and bipolar disorder

The pathogenic processes involved in the increase prevalence of WMH in BD patients are likely to be multiple and are certainly not immune to the effect of psychiatric medication and lifestyle factors, including drug abuse and tobacco. Despite this, there is an excessive burden of these lesions that needs to be properly clarified, so that an early intervention and prevention can be designed.

Functional and structural abnormalities of prefrontal cortex and limbic structures have been consistently reported in BD, supporting the hypothesis of a dysfunction of neural circuits related to emotions, reward, and cognitive processing in this disease (34). Stress and anxiety are important features of BD, emerging early and persisting throughout the disease (34). Stress and anxiety have been associated with elevation of peripheral inflammatory biomarkers, reflecting a dysregulation of the immune response (35). An inflammatory status has been consistently reported in both adults and adolescents with BD (5). Patients with BD have a high occurrence of inflammatory and auto-immune disorders, corroborating the hypothesis of involvement of inflammatory dysregulation in the pathogenesis of the disease (35). The dysregulation of the inflammation appears to have neurotoxic effects, and has been linked to widespread abnormalities of the white matter microstructure (36). The increased oxidative stress and excitotoxicity are potential mechanism of neurotoxic effects of inflammation (34). Increased oxidative stress was reported in postmortem studies of bipolar patients, especially in frontal regions (5).An emerging pathway potentially mediating neurotoxic effects of inflammation in BD is the tryptophan-kynurenine pathway (34).

Inflammation is an important factor of vascular system health, being involved in the initiation and progress of atherosclerosis (5). The interaction between reactive oxygen-species and the endothelium-derived nitric oxide leads to endothelial dysfunction, and consequent endothelium damage (5). Endothelial dysfunction and inflammation are thought to be involved in the pathogenesis of CSVD (37). Endothelial dysfunction is also associated with increased risk of CVRF, including hypertension and diabetes (38), which are important risk factors for CSVD (3).

The Brain-derived neurotrophic factor (BDNF) is an important neurotrophin for angiogenesis and revascularization (5). Reduced BDNF levels contribute to compromised endothelial integrity and endothelial cells apoptosis (5).

Decreased levels of BDFN levels during symptomatic episodes of BD have been reported in several studies (5). BDNF deficiency in BD may, therefore, be implicated in damage to cerebral vessels damage, an thus CSVD (5).

These dysfunctions of inflammatory status, oxidative stress and BDNF levels in BD may mediate CSVD, resulting in an increase of WMH (5).

### 4.2 Limitations

The use of small samples and the lack of studies in community cohorts limits statistical power and generalizability of the results. A systematic problem to be considered in this type of studies is the underrepresentation of cases, as some patients are unable to undergo MRI scan. Furthermore, almost half of the studies did not mention or select community controls, and none reported having independent validation in the selection process.

Sex differences could not be tested because some studies, Dupont RM et al. (12) and Aylward EH et al. (17), did not provide this information. This is an important limitation as evidence reports higher prevalence of WMH in women (4).

Age is also a crucial factor influencing the prevalence of WMH, but the studies covered a wide age range without carrying out sub-analysis with participants grouped by age (2). While there is published research of WMH in children and adolescents, our review focused on adults. Published research show mixed results (39–42) and a recent meta-analysis reported no difference in prevalence in adolescents (43).

Cardiovascular risk factors strongly influence the prevalence of WMH (2), but not all studies measured or controlled for this confounder. Most studies did not also evaluate or control for psychiatric medication use, and substance use disorders. Many psychiatric medication, including some antipsychotics, mood stabilizers and antidepressants, are associated with cardiometabolic risk factors and increased blood pressure (44), and substance use disorders are considered a risk for cardiovascular disorders (45). The lack of identification of CVRF, use of psychiatric medication and substance use disorders by a part of the studies is an important limitation, as it makes it impossible to measure the effect of these variables.

We found moderate heterogeneity in the reported prevalence of WMH across studies, which is considered a main limitation. The sample size, publication year, mean age of the sample and field strength have shown not to be significant sources of heterogeneity. Yet, when performing a separate analysis with 0.5-T vs. 1.5-T field strength, we only found differences between BD and controls in studies using 1.5-T. This suggests that field strength of MRI may influence the study results. Field strength is known to increase the quality of the image (46) and 1.5-T has been shown to have superior depiction of pathology compared to 0.5-T field strength (47, 48). As expected, 3-T scans have been shown to have greater conspicuity for detection of white matter lesions than 1.5-T (49). The use of low filed strength sequences is a limitation. Another limitation related to the acquisition characteristics is the impossibility of analyzing subgroups by slice thickness. Other parameters varied between studies using the same slice thickness, namely interslice gap, echo time, repetition time and processing software. In addition to the slice thickness, these other sequence parameters contribute to the discriminative power of the images. Furthermore, the echo and repetition time data are missing in Dupont RM et al. (19), Sassi RB et al. (24), Silverstone T et al. (25). Imaging sequence parameters affect image quality and artifacts, which can influence the identification of WMH (50, 51). Also, several different scanners were used. Differences related to the electrical system, Faraday cage and channels are a source of noise that can introduce variability. Combining images from different scanners can be a cause of systematic errors. However, the potential of confounding effects introduced using different scanners remains unknown (52).

BD type have been described as having impact the white matter (32, 53). Because information on BD clinical features is missing, a subgroup analysis for BD type could not be performed.

Lastly, a limitation of this study is that the search was restricted to two electronic databases.

### 4.3 Agreements and disagreements with other studies

There are four published meta-analyses which have reviewed the prevalence of WMH in BD (9, 18, 54, 55). Three studies reported a significant difference in the prevalence of WMH in BD patients compared to controls, with an estimated OR of 3 (95% CI 1.94, 5.62 (18)), 3.29 (95% CI 2.14, 5.07 (54)) and 2.5 (95% CI 1.9, 3.3 (9)).

The study from Kempton MJ et al. (55) found significant increases in deep WMH, reporting an OR of 2.49 (95% CI 1.64, 3.79), but not in periventricular WMH in BP patients. The oldest reviews (18, 54) reported non-significant heterogeneity across studies while in the more recent reviews the heterogeneity was significant (9, 55).

### 4.4 Location of hyperintensities

Research consistently reports frontal and fronto-parietal location of WMH. Hyperintensities are neuroimaging correlates of pathological changes associated with brain tissue damage, thus interfering with brain connectivity of frontolimbic circuits involving the prefrontal cortex, medial temporal lobe and striatum (regions anatomically related to the pathophysiology of BD) (56). This may result in decreased prefrontal modulation of the anterior limbic network and mood dysregulation (57, 58).

### 4.5 Quality of evidence

We graded the evidence for the outcomes as fair.

### 4.6 Potential biases in the review process

We have applied language restrictions, but the literature search is unlikely to have missed relevant case-control studies. The publications spanned over a wide time range (from 1990 to 2022), leading to heterogeneous methods and data reporting practices. For older publications the authors could not be reached, resulting in lack of information for assessment of risk of bia

## 5 Conclusion

We found evidence that BD patients have a higher risk of having WMH, and so of having CSVD, compared to healthy controls.

This result strongly suggests that CSVD and BD share common pathophysiological processes which warrant further research. It remains to be determined if a stricter control of vascular risk factors in these patients can prevent the onset and/or delay the natural course of BD.

## Data availability statement

The original contributions presented in the study are included in the article/**Supplementary Material**. Further inquiries can be directed to the corresponding author.

## Author contributions

TS: Conceptualization, Data curation, Formal analysis, Investigation, Methodology, Project administration, Resources, Software, Supervision, Validation, Visualization, Writing – original draft, Writing – review & editing. CN: Methodology, Software, Validation, Visualization, Writing – review & editing. AR: Data curation, Investigation, Writing – review & editing. IS: Conceptualization, Data curation, Investigation, Project administration, Supervision, Validation, Writing – review & editing. JC: Conceptualization, Funding acquisition, Investigation, Resources, Supervision, Writing – review & editing.
