# Supplementary material for: White matter hyperintensities in bipolar disorder: systematic review and meta-analysis
Source: Front Psychiatry. 2024 Jan 26;15:1343463. doi: 10.3389/fpsyt.2024.1343463 (PMC10853814; doi:10.3389/fpsyt.2024.1343463)
Supplement: Supplementary file 1 [file Table_1.docx]

Supplementary material 1. Research question – People, Exposure, Outcome (PEO).

| Research question | Do adults with Bipolar disorder have higher prevalence of WMH than healthy controls? |
| --- | --- |
| Population | Adult patients |
| Exposure | Bipolar disorder |
| Outcome | White matter hyperintensities |
