# Supplementary material for: White matter hyperintensities in bipolar disorder: systematic review and meta-analysis
Source: Front Psychiatry. 2024 Jan 26;15:1343463. doi: 10.3389/fpsyt.2024.1343463 (PMC10853814; doi:10.3389/fpsyt.2024.1343463)
Supplement: Supplementary file 2 [file Table_2.docx]

Supplementary material 3. Research strategy

| Date of search: May 20, 2023**;** Limits: English, Portuguese, and Spanish | |
| --- | --- |
| **PUBMED/MEDLINE** | |
| **(((((White matter hyperintensities) OR (Cerebral Small Vessel Diseases)) OR (microvascular disease)) OR (leukoaraiosis)) OR (Leukoencephalopathy)) AND ((bipolar disorder) OR (mania) OR (hypomania))))** | 178 articles retrieved |
| **EMBASE** | |
| **(((((White matter hyperintensities) OR (Cerebral Small Vessel Diseases)) OR (microvascular disease)) OR (leukoaraiosis)) OR (Leukoencephalopathy)) AND ((bipolar disorder) OR (mania) OR (hypomania))))** | 242 articles retrieved |
