# Supplementary material for: White matter hyperintensities in bipolar disorder: systematic review and meta-analysis
Source: Front Psychiatry. 2024 Jan 26;15:1343463. doi: 10.3389/fpsyt.2024.1343463 (PMC10853814; doi:10.3389/fpsyt.2024.1343463)
Supplement: Supplementary file 3 [file Table_3.docx]

Supplementary material 4. Detailed table of Newcastle- Ottawa quality assessment scale for selection category.

|  | **SELECTION CATEGORY** | | | |
| --- | --- | --- | --- | --- |
| **STUDY** | **1.Is the case definition adequate?** | **2.Representativeness of the cases.** | **3.Selection of controls** | **4.Definition of controls** |
| Dupont RM et al., (1990)(11) | No information of independent validation. | Not satisfying requirements. | No representative community controls. | *Controls had no history of bipolar disorder. |
| Swayze VW et al., (1990)(12) | No information of independent validation. | Not satisfying requirements. | No representative community controls. | *Controls had no history of bipolar disorder. |
| Figiel GS et al., (1991)(23) | No information of independent validation. | Not satisfying requirements. | No representative community controls. | *Controls had no history of bipolar disorder. |
| McDonald WM et al., (1991) | No information of independent validation. | Not satisfying requirements. | No representative community controls. | *Controls had no history of bipolar disorder. |
| Strakowski SM et al., (1993)(26) | No information of independent validation. | Not satisfying requirements. | No representative community controls. | *Controls had no history of bipolar disorder. |
| Aylward EH et al., (1994)(27) | No information of independent validation. | Not satisfying requirements. | No representative community controls. | *Controls had no history of bipolar disorder. |
| Altshuler LL et al., (1995)(28) | No information of independent validation. | *Patients consecutively admitted to inpatient and outpatient units. | No representative community controls. | *Controls had no history of bipolar disorder |
| Dupont RM et al., (1995)(29) | No information of independent validation. | Not satisfying requirements. | No representative community controls. | *Controls had no history of bipolar disorder |
| Persaud R et al., (1997)(30) | No information of independent validation. | Not satisfying requirements. | No representative community controls. | *Controls had no history of bipolar disorder |
| McDonald WM et al., (1999) | Record evaluation. | Not satisfying requirements. | No representative community controls. | *Controls had no history of bipolar disorder |
| Krabbendam L et al., (2000)(13) | No information of independent validation. | Not satisfying requirements. | No representative community controls. | *Controls had no history of bipolar disorder |
| Moore PB et al., (2001)(14) | No information of independent validation. | Not satisfying requirements. | No representative community controls. | *Controls had no history of bipolar disorder |
| Sassi RB et al., (2003)(15) | No information of independent validation. | Not satisfying requirements. | No description on controls recruitment. | *Controls had no history of bipolar disorder |
| Silverstone T et al., (2003)(16) | No information of independent validation. | Not satisfying requirements. | No description on controls recruitment. | *Controls had no history of bipolar disorder |
| Ahn KH et al., (2004)(17) | No information of independent validation. | Not satisfying requirements. | No representative community controls. | *Controls had no history of bipolar disorder |
| El-Badri SM et al., (2006)(18) | No information of independent validation. | Not satisfying requirements. | No description on controls recruitment. | *Controls had no history of bipolar disorder |
| Gulseren S et al., (2006)(19) | No information of independent validation. | Not satisfying requirements. | No description on controls recruitment. | *Controls had no history of bipolar disorder |
| Tamashiro et al., (2008)(20) | No information of independent validation. | Not satisfying requirements. | No description on controls recruitment. | *Controls had no history of bipolar disorder |
| Lloyd AJ et al., (2009)(32) | No information of independent validation. | Not satisfying requirements. | No representative community controls. | *Controls had no history of bipolar disorder |
| Macritchie KA et al., (2010)(21) | No information of independent validation. | Not satisfying requirements. | No description on controls recruitment. | *Controls had no history of bipolar disorder |
| Kieseppä T et al., (2014)(22) | No information of independent validation. | Not satisfying requirements. | *Representative community controls. | *Controls had no history of bipolar disorder |
| Kieseppä T et al., (2022)(24) | No information of independent validation. | Not satisfying requirements. | *Representative community controls. | *Controls had no history of bipolar disorder |
