# Supplementary material for: White matter hyperintensities in bipolar disorder: systematic review and meta-analysis
Source: Front Psychiatry. 2024 Jan 26;15:1343463. doi: 10.3389/fpsyt.2024.1343463 (PMC10853814; doi:10.3389/fpsyt.2024.1343463)
Supplement: Supplementary file 4 [file Table_4.docx]

Supplementary material 5. Detailed table of Newcastle- Ottawa quality assessment scale for comparability and exposure categories.

|  |  | **EXPOSURE CATEGORY** | | |
| --- | --- | --- | --- | --- |
| **STUDY** | **COMPARABILITY CATEGORY** | **1.Ascertainment of exposure** | **2.Same method of ascertainment** | **3.Non-response rate** |
| Dupont RM et al., (1990)(11) | Matched for age but not for sex. | *Senior neuroradiologist blind to the group. | *Same acquisition protocol. | No information. |
| Swayze VW et al., (1990)(12) | *Matched for age and sex. | *Team of 2 radiologists blind to the diagnosis. | *Same acquisition protocol. | No information. |
| Figiel GS et al., (1991)(23) | *Matched for age and sex. | *Neuroradiologist blind to the group. | *Same acquisition protocol. | No information. |
| McDonald WM et al., (1991) | *Matched for age and sex. | *Team of 2 ratters blind to the diagnosis. | *Same acquisition protocol. | No information. |
| Strakowski SM et al., (1993)(26) | *Matched for age and sex. | *Senior board neuroradiologist and another rater; both blind to the diagnosis. | *Same acquisition protocol. | No information. |
| Aylward EH et al., (1994)(27) | Matched for age but no information concerning sex match. | *Neuroradiologist blind to the diagnosis. | *Same acquisition protocol. | No information. |
| Altshuler LL et al., (1995)(28) | *Matched for age and sex. | *Neuroradiologist blind to the diagnosis. | *Same acquisition protocol. | No information. |
| Dupont RM et al., (1995)(29) | *Matched for age and sex. | *Neuroradiologist blind to the diagnosis. | *Same acquisition protocol. | No information. |
| Persaud R et al., (1997)(30) | *Matched for age and sex. | *Neuroradiologist blind to the diagnosis. | *Same acquisition protocol. | No information. |
| McDonald WM et al., (1999) | *Matched for age and sex. | *Neuroradiologist blind to the diagnosis. | Variable acquisition protocol. | No information. |
| Krabbendam L et al., (2000)(13) | *Matched for age and sex. | *Neuroradiologist blind to the diagnosis. | *Same acquisition protocol. | No information. |
| Moore PB et al., (2001)(14) | *Matched for age and sex. | *Two examinators blind to the diagnosis | *Same acquisition protocol. | No information. |
| Sassi RB et al., (2003)(15) | *Matched for age and sex. | *Two ratters blind to the diagnosis. | *Same acquisition protocol. | No information. |
| Silverstone T et al., (2003)(16) | *Matched for age and sex. | *Two radiologists blind to the diagnosis. | *Same acquisition protocol. | No information. |
| Ahn KH et al., (2004)(17) | *Matched for age and sex. | *Neuroradiologist blind to the diagnosis. | *Same acquisition protocol. | No information. |
| El-Badri SM et al., (2006)(18) | *Matched for age and sex. | *Neuroradiologist blind to the diagnosis. | *Same acquisition protocol. | No information. |
| Gulseren S et al., (2006)(19) | *Matched for age and sex. | Assessment by a radiologist; not blind to the diagnosis? | *Same acquisition protocol. | No information. |
| Tamashiro et al., (2008)(20) | *Matched for age and sex. | *Neuroradiologist blind to the diagnosis. | *Same acquisition protocol. | No information. |
| Lloyd AJ et al., (2009)(32) | *Matched for age and sex. | *Two ratters blind to the diagnosis. | *Same acquisition protocol. | No information. |
| Macritchie KA et al., (2010)(21) | *Matched for age and sex. | *Three ratters blind to the diagnosis. | *Same acquisition protocol. | No information. |
| Kieseppä T et al., (2014)(22) | *Matched for age and sex. | *Two radiologists blind to the diagnosis. | *Same acquisition protocol. | No information. |
| Kieseppä T et al., (2022)(24) | *Matched for age and sex. | *Two radiologists blind to the diagnosis. | *Same acquisition protocol. | No information. |
