# Supplementary material for: White matter hyperintensities in bipolar disorder: systematic review and meta-analysis
Source: Front Psychiatry. 2024 Jan 26;15:1343463. doi: 10.3389/fpsyt.2024.1343463 (PMC10853814; doi:10.3389/fpsyt.2024.1343463)
Supplement: Supplementary file 5 [file Table_5.docx]

Supplementary material 6. Methods used in the studies for major confounding factors controlling. *CVRF - cardiovascular risk factors.*

| **Study** | **Age** | **Sex** | **CVRF** | **Psychiatric medication** | **Substance abuse** | **Medical/neurological comorbidity** |
| --- | --- | --- | --- | --- | --- | --- |
| Dupont RM et al., (1990)(11) | Age match | Not controlled | Not controlled | No controlled. | Restriction in participant selection | Restriction in participant selection |
| Swayze VW et al., (1990)(12) | Age match | Sex match | Not controlled | Not controlled | Not controlled | No controlled. |
| Figiel GS et al., (1991)(23) | Age match | Sex match | Demonstrated balance between groups for the confounder | Not controlled | Not controlled | Restriction in participant selection |
| McDonald WM et al., (1991) | Age match | Sex match | Not controlled | Not controlled | Not controlled | Restriction in participant selection |
| Strakowski SM et al., (1993)(26) | Age match | Sex match | Not controlled | Restriction in participant selection | Not controlled | Restriction in participant selection |
| Aylward EH et al., (1994)(27) | Age match | Not controlled | Not controlled | Not controlled | Restriction in participant selection | Restriction in participant selection |
| Altshuler LL et al., (1995)(28) | Age match | Sex match | CVRF match | Not controlled | Not controlled | Restriction in participant selection |
| Dupont RM et al., (1995)^a^(29) | Age match | Sex match | Restriction in participant selection | Not controlled | Restriction in participant selection. | Restriction in participant selection. |
| Persaud R et al., (1997)(30) | Age match | Sex match | Restriction in participant selection | Not controlled | Restriction in participant selection. | Not controlled |
| McDonald WM et al., (1999) | Age match | Sex match | Not controlled | Not controlled | Not controlled | Not controlled |
| Krabbendam L et al., (2000)(13) | Age match | Sex match | Restriction in participant selection | Not controlled | Restriction in participant selection. | Restriction in participant selection. |
| Moore PB et al., (2001)(14) | Age match | Sex match | Not controlled | Not controlled | Restriction in participant selection. | Restriction in participant selection. |
| Sassi RB et al., (2003)(15) | Age match | Sex match | Not controlled | Restriction in participant selection | Restriction in participant selection. | Restriction in participant selection. |
| Silverstone T et al., (2003)(16) | Age match | Sex match | Restriction in participant selection | Not controlled | Not controlled | Restriction in participant selection. |
| Ahn KH et al., (2004)(17) | Age match | Sex match | Not controlled | Not controlled | Restriction in participant selection. | Restriction in participant selection. |
| El-Badri SM et al., (2006)(18) | Age match | Sex match | Not controlled | Not controlled | Restriction in participant selection. | Restriction in participant selection. |
| Gulseren S et al., (2006)(19) | Age match | Sex match | Restriction in participant selection | Not controlled | Restriction in participant selection | Restriction in participant selection. |
| Tamashiro et al., (2008)(20) | Age match | Sex match | Demonstrated balance between groups for the confounder | Not controlled | Demonstrated balance between groups for the confounder | Restriction in participant selection. |
| Lloyd AJ et al., (2009)(32) | Age match | Sex match | Restriction in participant selection | Not controlled | Restriction in participant selection | Restriction in participant selection. |
| Macritchie KA et al., (2010)(21) | Age match | Sex match | Not controlled | Not controlled | Restriction in participant selection | Restriction in participant selection. |
| Kieseppä T et al., (2014)(22) | Age match | Sex match | Restriction in participant selection | Not controlled | Restriction in participant selection. | Restriction in participant selection. |
| Kieseppä T et al., (2022)(24) | Age match | Sex match | Restriction in participant selection | Not controlled | Restriction in participant selection. | Restriction in participant selection. |
