# Supplementary material for: White matter hyperintensities in bipolar disorder: systematic review and meta-analysis
Source: Front Psychiatry. 2024 Jan 26;15:1343463. doi: 10.3389/fpsyt.2024.1343463 (PMC10853814; doi:10.3389/fpsyt.2024.1343463)
Supplement: Supplementary file 6 [file Table_6.docx]

Supplementary material 7. Meta-regression analysis of sample size as source of heterogeneity.

|  | Point estimate | Standard error | T | p-Value | 95% CI  Lower | 95% CI  upper |
| --- | --- | --- | --- | --- | --- | --- |
| Intercept | 1.540 | 0.611 | 2.518 | 0.020 | 0.264 | 2.816 |
| Sample size | -0.006 | 0.008 | -0.802 | 0.432 | -0.023 | 0.010 |
