# Supplementary material for: White matter hyperintensities in bipolar disorder: systematic review and meta-analysis
Source: Front Psychiatry. 2024 Jan 26;15:1343463. doi: 10.3389/fpsyt.2024.1343463 (PMC10853814; doi:10.3389/fpsyt.2024.1343463)
Supplement: Supplementary file 7 [file Table_7.docx]

Supplementary material 8. Meta-regression analysis of publication year as source of heterogeneity.

|  | Point estimate | Standard error | T | p-Value | 95% CI  Lower | 95% CI  upper |
| --- | --- | --- | --- | --- | --- | --- |
| Intercept | 42.382 | 66.451 | 0.637 | 0.530 | -96.233 | 180.997 |
| Publication year | -0.020 | 0.033 | -0.621 | 0.541 | -0.089 | 0.048 |
