# Supplementary material for: White matter hyperintensities in bipolar disorder: systematic review and meta-analysis
Source: Front Psychiatry. 2024 Jan 26;15:1343463. doi: 10.3389/fpsyt.2024.1343463 (PMC10853814; doi:10.3389/fpsyt.2024.1343463)
Supplement: Supplementary file 8 [file Table_8.docx]

Supplementary material 9. Meta-regression analysis of mean age as source of heterogeneity.

|  | Point estimate | Standard error | T | p-Value | 95% CI  Lower | 95% CI  upper |
| --- | --- | --- | --- | --- | --- | --- |
| Intercept | 0.463 | 1.076 | 0.43 | 0.672 | -1.782 | 2.71 |
| Total mean age | 0.015 | 0.025 | 0.595 | 0.559 | -0.037 | 0.067 |
