# Supplementary material for: White matter hyperintensities in bipolar disorder: systematic review and meta-analysis
Source: Front Psychiatry. 2024 Jan 26;15:1343463. doi: 10.3389/fpsyt.2024.1343463 (PMC10853814; doi:10.3389/fpsyt.2024.1343463)
Supplement: Supplementary file 9 [file Table_9.docx]

Supplementary material 10. Meta-regression analysis of field strength as source of heterogeneity.

|  | Point estimate | Standard error | T | p-Value | 95% CI  Lower | 95% CI  upper |
| --- | --- | --- | --- | --- | --- | --- |
| Intercept | 4.007 | 1.773 | 2.259 | 0.036 | 0.295 | 7.720 |
| 0.5-T field | -3.116 | 1.827 | -1.705 | 0.104 | -6.941 | 0.708 |
| 1.5-T field | -2.952 | 1.795 | -1.644 | 0.117 | -6.710 | 0.807 |
